# Plasma MicroRNA Expression Profiles in Psoriasis

**DOI:** 10.1155/2020/1561278

**Published:** 2020-01-16

**Authors:** Shiju Xiao, Xin Liu, Xiaoxu Wang, Hongpeng Lv, Junbo Zhao, Xinwei Guo, Fuyang Xian, Yunrun Ji, Guangzhong Zhang

**Affiliations:** ^1^Beijing Hospital of Traditional Chinese Medicine, Capital Medical University, Beijing 100010, China; ^2^Graduate School, Capital Medical University, Beijing 100069, China; ^3^Beijing Institute of Traditional Chinese Medicine, Beijing 100010, China; ^4^Beijing University of Chinese Medicine, Beijing 100029, China; ^5^Beijing Daxing District Hospital of Integrated Traditional Chinese and Western Medicine, Beijing 100076, China

## Abstract

**Background:**

Psoriasis is an immune-mediated inflammatory chronic skin disease characterized by chronic inflammation in the dermis, parakeratosis, and excessive epidermal growth. MicroRNAs (miRNAs) are key regulators of immune responses. Although differential expression of miRNAs has been reported in certain inflammatory autoimmune diseases, their role in psoriasis has not been fully illuminated. Our aims were to confirm plasma miRNA expression signatures in psoriasis and to examine their potential influence on psoriasis pathogenesis.

**Methods:**

A miRNome PCR array was used to analyse the plasma of psoriasis patients and healthy donors. We performed miRNA pathway enrichment and target gene network analyses on psoriasis plasma samples.

**Results:**

We found several specific plasma miRNA signatures relevant to psoriasis. The miRNAs targeted pathways associated with psoriasis, such as the VEGF, MAPK, and WNT signaling pathways. Network analysis revealed pivotal deregulated plasma miRNAs and their relevant target genes and pathways regulating psoriasis pathogenesis.

**Conclusions:**

This study analysed the expression of plasma miRNAs and their target pathways, elucidating the pathogenesis of psoriasis; these results may be used to design novel therapeutic strategies and to identify diagnostic biomarkers for psoriasis.

## 1. Introduction

Psoriasis is an immune-mediated inflammatory chronic skin disease characterized by chronic inflammation in the dermis, parakeratosis, and excessive epidermal growth [1]. Skin lesions of psoriasis are characterized by infiltration of inflammatory cells and abnormal differentiation and hyperproliferation of keratinocytes [1]. Psoriasis affects 2-3% of the global population and seriously affects the quality of life of patients [2, 3]. There are 4 types of psoriasis, including psoriasis vulgaris, pustular psoriasis, psoriatic arthritis, and erythrodermic psoriasis [4]. However, the pathogenesis of psoriasis is still poorly understood, as psoriasis is a disease influenced by many different factors [4]. It is widely accepted that genetic susceptibility, cell cycle, immunity, inflammation, and neurotransmitters are involved [5–7]. Recently, abnormal genetic and environmental elements, particularly deregulated microRNAs (miRNAs) and their associated genes, have been indicated to be causative factors of psoriasis [8]. However, only a limited number of genes have been detected [9]. Currently, psoriasis is mainly diagnosed by clinical features (morphological evaluation of skin lesions). On occasion, a dermatopathologic evaluation may be helpful to confirm the diagnosis of psoriasis. However, unlike other autoimmune diseases, a histopathological examination and blood tests are generally not valuable tools to diagnose psoriasis, so diagnostic tests are almost unavailable [10].

The psoriatic lesion is characterized by T cell infiltration, increased chemokines, and angiogenesis, which may boost skin inflammation [11]. Increased Th17, CD4^+^, and CD8^+^ T cells and the interleukin- (IL-) 17 and IL-23 cytokines have been found in psoriatic lesions and peripheral blood, suggesting the involvement of innate and adaptive immunity in the pathogenesis of psoriasis [12, 13].

Currently, no specific markers that can help diagnose psoriasis and predict disease progression and remedial effects are found. Thus, a biomarker that can distinguish clinical types of psoriasis or can be used as a predictive biomarker for psoriasis progression is needed.

MicroRNAs (miRNAs) are small noncoding RNAs, approximately 22 to 25 nucleotides in length on average, with important roles in posttranscriptional gene expression. Deregulation of miRNAs and the corresponding target gene expression have been shown to be involved in psoriasis pathogenesis [14, 15]. miRNAs play a critical role in various autoimmune diseases, including psoriasis [16–18]. Recently, the number of miRNAs involved in immune system function and development has increased remarkably, and there has been a wide-ranging discussion of their possible use in therapies for immunological diseases [19]. miRNAs can regulate the proliferation, differentiation, and cytokine response of keratinocytes, the activation and survival of T cells, and the crosstalk between immunocytes and keratinocytes through the regulation of chemokine production in psoriasis. Recently, it has become evident that genetic polymorphisms in miRNA genes and/or in miRNA-binding sites of target genes can affect miRNA activity and contribute to disease susceptibility [20]. The concept that miRNAs take part in the pathogenesis of diseases, especially refractory diseases with unknown mechanisms, might lead to a new efficacious treatment. These studies emphasized the profound implication of miRNAs as regulatory molecules in autoimmunity and the intriguing possibility of using miRNAs as disease biomarkers in immunological diseases.

Studies have examined the role of miRNAs identified from human psoriatic skin, blood, and hair samples in relation to psoriasis pathogenesis, diagnosis, and treatment [4, 21]. Genetic polymorphisms related to specific miRNAs, such as miR-146a, are associated with psoriasis susceptibility [4]. Key roles of several unique miRNAs, such as miR-203 and miR-125b, in inflammatory responses and immune dysfunction, as well as hyperproliferative disorders of psoriatic lesions, have been revealed [22–24]. Moreover, circulating miRNAs detected from blood samples have the potential to be used in clinical applications as biomarkers of diagnosis, prognosis, and treatment responses [4]. These works underscored the potential importance of miRNAs in the diagnosis, prognosis, and treatment of psoriasis. However, further study in this field is needed, as the exact roles of miRNAs in psoriasis have not been fully elucidated. High-throughput miRNA expression studies have been performed to confirm plasma miRNAs specifically related to psoriasis [11, 24–26], but few studies have conducted a network analysis of plasma microRNAs and their target genes in psoriasis patients.

We performed a miRNome PCR array analysis on the plasma of psoriasis patients. Distinct plasma miRNA signatures relevant to psoriasis compared to healthy controls were confirmed. Pathway enrichment analysis of target genes of deregulated miRNAs revealed signaling pathways closely related to psoriasis pathogenesis, such as the VEGF signaling cascades, mitogen-activated protein (MAP) kinase pathway, and wingless-related integration site (WNT) pathway.

The network analysis of the interactions between plasma miRNAs and target genes/pathways in psoriasis illustrated psoriasis pathogenesis. This study illuminated some potential mechanisms of psoriasis pathogenesis by analysing deregulated plasma miRNAs, which may be used for the design of novel therapeutic strategies and diagnostic biomarkers of psoriasis.

## 2. Materials and Methods

### 2.1. Patients and Controls

Fifteen clinically diagnosed psoriasis patients (9 males and 6 females) and four healthy control subjects were included in our research. The psoriasis patients had not received systemic immunosuppressive medications for at least 1 month prior to study participation. The study was approved by the Ethical Committee of the Beijing Hospital of Traditional Chinese Medicine. Written informed consent was obtained from all the participants in the study. This research was performed in adherence to the principles expressed in the Helsinki Declaration.

### 2.2. Sample Preparation and RNA Extraction

Whole blood samples of healthy controls and psoriasis patients were collected using ethylenediaminetetraacetic acid- (EDTA-) containing tubes. All blood samples were centrifuged at 3,000 g for 20 min at 4°C. We collected the supernatants and then centrifuged again (13,000 g for 15 min at 4°C) to remove the cellular components. Finally, the purified plasma samples were obtained and stored at -70°C until further processing. One millilitre of each sample was subjected to RNA extraction.

Total RNA from each plasma sample was extracted using TRIzol-LS Reagent (Invitrogen Life Technologies) according to the manufacturer's instructions. The concentration and purity of RNA was determined with NanoDrop ND-1000.

### 2.3. miRNA Profiling by Exiqon miRNA qPCR Panel

According to the RT^2^ RNA QC PCR Array Protocol, RNA isolation (including homogenization, phase separation, RNA precipitation, RNA washing, RNA elution, and isolation of small-quantity RNA), RNA yield and quality assessment (including UV absorbance and denaturing agarose gel electrophoresis), cDNA synthesis (including diluting template RNA, preparing reagents, combining reagents, vortexing and centrifuging reagents, and incubating and heat inactivating reagents), and real-time PCR (including preparing reagents, diluting cDNA template 80x in nuclease-free water, combining reagents, vortexing and centrifuging reagents, and real-time PCR amplification) were performed by Exiqon miRCURY-Ready-to-Use PCR-Human-panel-I+II-V1.M (Exiqon miRNA qPCR panel, Vedbaek, Denmark), which could detect 764 miRNAs in plasma to identify differentially expressed miRNAs on the ABI PRISM 7900 system (Applied Biosystems) [27, 28]. Real-time PCR amplification was followed by melting curve analysis. Melting curve analysis of the PCR product(s) was performed to verify their specificity and identity. The relative expression between psoriasis patients and normal controls was estimated by the 2^−ΔΔCt^ method. We calculated the ΔΔCt for each gene across two groups using the following equations, where group 1 is the control group and group 2 is the experimental group. Finally, we calculated the fold change for each gene from group 1 to group 2 by 2^−ΔΔCt^.

(1) $$\begin{array}{c} \Delta \text{Ct }\left(\text{group }1\right)=\text{average Ct}–\text{average of housekeeping gene}{\text{s}}^{\text{'}}\text{ Ct for group }1\text{ array},\\ \Delta \text{Ct }\left(\text{group }2\right)=\text{average Ct}–\text{average of housekeeping gene}{\text{s}}^{\text{'}}\text{ Ct for group }2\text{ array},\\ \Delta \Delta \text{Ct}=\Delta \text{Ct}\left(\text{group }2\right)-\Delta \text{Ct }\left(\text{group }1\right). \end{array}$$

### 2.4. miRNA Target Gene Prediction

Screening of differential plasma miRNA was performed by the TwoClassDif database, and hierarchical clustering was performed by EPCLUST [29, 30]. The RVM *t*-test was applied to filter the differentially expressed plasma miRNAs for the control and experimental groups because the RVM *t*-test can raise degrees of freedom effectively in the cases of small samples. After the significant analysis and FDR analysis, we selected the differentially expressed plasma miRNAs according to the *P* value threshold. A *P* value < 0.05 was considered to have a significant difference.

MicroRNA target genes were predicted using the following miRNA target prediction tools: miRanda (August 2010 release) [31] and TargetScan (http://www.targetscan.org/vert_71/) [32]. Only genes predicted by both databases were chosen as conjectural miRNA targets for pathway analysis.

### 2.5. Pathway Enrichment and Network Analysis of MicroRNA Targets

Gene Ontology (GO) (http://www.geneontology.org) is a widely used database for functional enrichment analysis, which is helpful for understanding the functions of genes and proteins. GO analysis was applied to analyse the main function of the differentially expressed genes according to the GO database, which is the key functional classification system of NCBI and can organize genes into hierarchical categories and uncover the gene regulatory network on the basis of biological processes and molecular functions [33, 34]. Enrichment provides a measure of the significance of the function: as the enrichment increases, the corresponding function is more specific; this correlation helps us find those GO terms with more concrete functional descriptions in the experiment.

Kyoto Encyclopedia of Genes and Genomes (KEGG) pathway analysis is an enrichment and network analysis of a gene function database, which helps researchers find deregulated biological signaling pathways [35]. Pathway analysis was used to determine the significant pathway of the differential genes according to KEGG, Biocarta, and Reactome. We turned to Fisher's exact test and the *χ*^2^ test to select the significant pathway, and the threshold of significance was defined by *P* value and FDR [35, 36].

In addition, the PathNet analysis is conducted, indicating the interacting relation between enriched pathways. The PathNet was the interaction net of the significant pathways of the differentially expressed genes and was built according to the interaction among pathways of the KEGG database to find the interaction among the significant pathways directly and systemically. It could summarize the pathway interaction of differentially expressed genes in diseases and determine the reason why certain pathways were activated [36, 37].

Moreover, intersecting genes of GO and KEGG were detected, and a miRNA-gene network was constructed. The relationship between plasma miRNAs and genes was counted by their differential expression values according to their interactions in the Sanger miRNA database, indicating the pivotal plasma miRNAs and genes in the pathogenesis of psoriasis [38]. A *P* value threshold of 0.05 and FDR correction were applied to the analysis.

### 2.6. Statistical Analysis

The relative quantification of plasma miRNAs was calculated by the equation 2^−ΔΔCt^. Data were expressed as the mean ± standard deviation (SD). A *P* value ≤ 0.05 was considered statistically significant. All statistical calculations were performed using SAS 9.4 software (Beijing Hospital of TCM Version, Order Number 9C1XJD).

## 3. Results

### 3.1. Global miRNA Expression Analysis of Plasma from Psoriasis Patients and Controls

**Figure 1:**
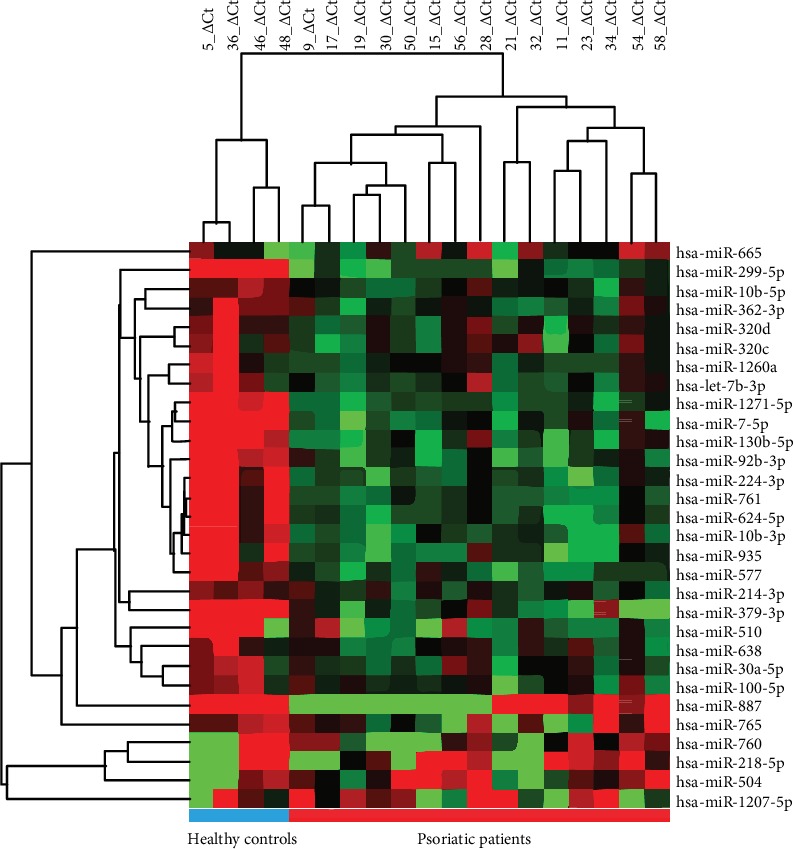
Hierarchical clustering of plasma samples. Heatmap showing expression of candidate miRNAs that are deregulated in the plasma of psoriasis patients compared with the plasma of normal controls (comparatively upregulated and downregulated miRNAs are indicated by red and green, respectively). The first 4 columns showed plasma from healthy individuals. The last 15 columns showed plasma from psoriasis patients. The lines indicate deregulated plasma miRNAs.

To identify plasma miRNA expression signatures associated with psoriasis, we analysed global miRNA expression profiles by the miRNome PCR array in plasma derived from 15 psoriasis patients and 4 healthy volunteers. The clinical characteristics of patients included in the miRNome PCR array study are reported in Table 1. In the pool of plasma miRNAs, we identified that 15 plasma miRNAs were upregulated and 15 plasma miRNAs were downregulated (*P* ≤ 0.05, fold change ≥ ∣2∣) in the plasma of psoriasis patients compared to healthy volunteers, showing a statistically significant difference between psoriasis patients and volunteers (Figure 1 and Table 2). Figure 1 shows that the colour of the patient group was different from that of the control group, which indicated the difference in plasma miRNA expression between the two groups.

### 3.2. Pathway Enrichment Analysis of Plasma miRNAs Deregulated in Psoriasis

To identify all the biological pathways targeted by deregulated plasma miRNAs in psoriasis, a pathway enrichment analysis based on annotated gene targets in GO was performed. This database can be used to evaluate miRNA regulatory action and to identify molecular pathways regulated by miRNAs. A functional pathway analysis using the KEGG pathway database was also performed. Genes targeted by deregulated miRNAs may be significant to psoriasis pathogenesis. Of note, a distinct enrichment of certain pathways was found; for example, pathways relevant to the immune system and proteoglycan metabolism and signaling pathways regulating apoptosis and/or the cell cycle were found [39].

**Figure 2:**
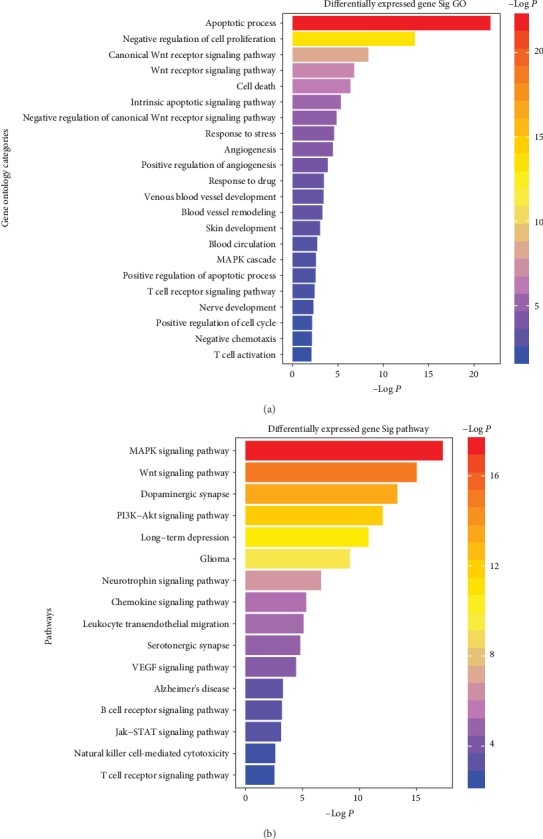
(a) GO-enriched biological process terms for the differentially expressed plasma miRNAs. (b) KEGG pathways enriched for differentially expressed plasma miRNAs.

In addition, many enriched pathways were found to be relevant to the multisystemic features of psoriasis.  Figure 2 graphically represents enriched pathways in psoriasis. The enriched pathways were associated with angiogenesis (i.e., angiogenesis, positive regulation of angiogenesis, blood vessel remodelling, blood circulation, VEGF signaling pathway, and PI3K-Akt signaling pathway), cell cycle (positive regulation of the cell cycle, cell death, and negative/positive regulation of cell proliferation), and apoptosis (apoptotic process, intrinsic apoptotic signaling pathway, and PI3K-Akt signaling pathways). Moreover, other associated enriched pathways in GO and KEGG databases were implicated in the immune response (i.e., T cell receptor signaling pathway, T cell activation, B cell receptor signaling pathway, leukocyte transendothelial migration, and natural killer cell-mediated cytotoxicity) and the inflammatory response (i.e., chemokine signaling pathway, negative chemotaxis, NF-kappa B signaling pathway, and JAK/STAT signaling pathway).

Moreover, the MAPK and WNT pathways were found to be significantly enriched in psoriasis patients (Figure 2). Psoriasis-associated plasma miRNAs target MAP kinases; it has been reported that miR-148a-3p targets MAPK1, MAP2K3, MAP3K4, and MAP4K3 [40–43]. Some miRNA target genes associated with the MAPK pathway were also found in our analysis: miR-320c and miR-320d were found to target MAPK1.

Interestingly, the pathways related to stress and nerves (central and peripheral) were found to be distinctly enriched in their responses, such as in their response to stress, neurotrophin signaling pathway, long-term depression, glioma, Alzheimer's disease, nerve development, dopaminergic synapse, and serotonergic synapse.

The “proteoglycans in cancer” pathway was overrepresented in the plasma miRNAs of the patients studied. These target genes include FGF1, WNT1, WNT10B, WNT11, WNT10A, and WNT4.

### 3.3. Network Analysis of Pathways Targeted by Deregulated Plasma miRNAs in Psoriasis

**Figure 3:**
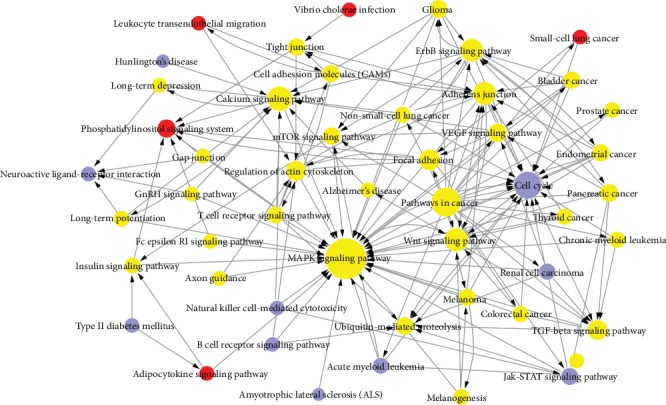
Network analysis of pathways targeted by deregulated plasma miRNAs in psoriasis (the red dots indicate the upregulated pathways, the blue dots indicate the downregulated pathways, and the yellow dots indicate the up/downregulated pathways).

The interactions of the pathways regulated by deregulated plasma miRNA target genes were determined by a network analysis including 49 pathway interactions constructed by KEGG (Figure 3).

Densely interconnected nodes expected to be associated with important biological processes of psoriasis pathogenesis were represented. The pathway network is graphically represented in Figure 3. The most enriched pathways, such as the MAPK, WNT, VEGF, and JAK/STAT signaling pathways; apoptosis; cell cycle; and pathways in cancer, were detected. Interestingly, the T cell receptor signaling pathway, B cell receptor signaling pathway, and natural killer cell-mediated cytotoxicity were significantly enriched. Moreover, the pathways related to long-term depression, Alzheimer's disease, and glioma were enriched.

### 3.4. Network Analysis of Deregulated Plasma miRNAs and Target Genes

**Figure 4:**
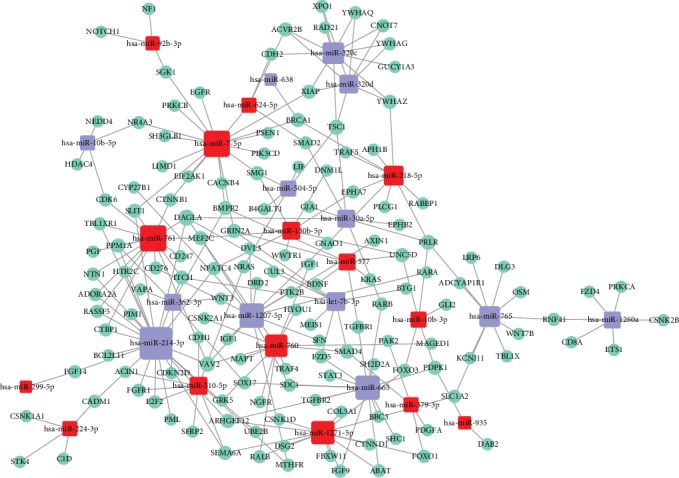
Network analysis of deregulated plasma miRNAs and target genes (the red squares indicate upregulated miRNAs, whereas the blue squares indicate downregulated miRNAs).

The relationship between the deregulated plasma miRNAs and the target genes in psoriasis was determined by network analysis. A microRNA-target gene network comprising 28 microRNAs and 144 target genes was constructed (Figure 4). The deregulated plasma miRNAs and targeted genes were relevant to biological processes confirmed in psoriasis pathogenesis, such as immune response, inflammation, angiogenesis, and apoptosis. Therefore, plasma microRNAs regulating genes relevant to psoriasis were identified.

Interestingly, some of the targeted genes were relevant to immune and inflammatory responses, such as IL4R. Moreover, upregulated plasma miRNA target genes associated with B cell receptor (BCR) (EGR3) and T cell receptor (TCR) (MLLT3, DUSP3, DUSP5, DUSP7, DUSP8, DUSP16, NR4A3, CD34, CD58, CD8A, CD247, and CD276) adaptive immune responses implicated in psoriasis were identified.

Moreover, target genes regulated by deregulated plasma miRNAs were also associated with the innate immune response, such as STAT2 (miR-214-3p and miR-665), STAT3 (miR-665), and genes belonging to the NF-kappa B pathway, including PLCG1 (miR-218-5p), PRKCB (miR-7-5p), CSNK2A1 (miR-760), and XIAP (miR-7-5p, miR-320c, and miR-320d). Notably, several miRNA-targeted genes were involved in the JAK/STAT signaling pathway that regulated PIK3R3, STAT3, JAK1, SOCS5, IL4R, IL11, JAK3, SOS1, and SOS2. The JAK/STAT signaling pathway is relevant to autoimmune diseases, which indicates that psoriasis is an autoimmune disease [44]. In addition, JAK/STAT signaling pathway inhibition is used for treating psoriasis [45].

Moreover, target genes of deregulated plasma miRNAs participated in angiogenesis; these target genes include PIK3CD, PIK3C2B, MAPK1, AKT2, and PIK3R3.

In addition, genes relevant to apoptosis were also targeted and include BCL2L11, PIK3CD, PIK3R3, PIK3C2B, MAP3K5, MAP3K11, and MAP4K4.

To detect the relationship between gene expression and deregulated plasma miRNAs, a network analysis was performed. The most upregulated miRNAs, such as miR-214-3p, miR-7-5p, miR-761, and miR-665, targeted significant pathways in psoriasis pathogenesis. The most targeted gene was DAGLA.

## 4. Discussion

Currently, systematic analyses of plasma miRNA expression profiles in psoriasis are being performed [4, 11, 22–26]. However, the potential roles of identified miRNAs in psoriasis have not been predicted with bioinformatics analysis, so further study is necessary in this field. Thus, a global miRNA expression profile in the plasma of psoriasis patients was determined to identify miRNAs associated with psoriasis, assisting in the analysis of potential pathways involved in psoriasis pathogenesis. Deregulated miRNA-targeted pathways, such as the inflammatory response, angiogenesis, apoptosis, and/or cell cycle pathways, indicated a corresponding impact on psoriasis.

The target genes were completely associated with representative characteristics of psoriasis. Some genes targeted by deregulated plasma miRNAs were associated with angiogenesis (i.e., VEGF pathway) and adaptive and innate immune responses, and proinflammatory genes, such as IL4R, were also identified. In addition, the JAK/STAT pathway, which is related to autoimmune diseases [44, 46], such as psoriasis [45, 47, 48], was also observed. Plasma miRNA target genes play a role in apoptosis, which is associated with psoriasis pathogenesis. AKT2 participates in the PI3K/AKT/mTOR signaling pathway as a critical mediator in psoriasis [49]. SDC2, a target of miR-665 and miR-1207-5p, was found to be upregulated in the blood and lesions of psoriasis patients and to be involved in angiogenesis and the migration and retention of leukocytes [39, 50].

Notably, the VEGF, MAPK, and WNT signaling pathways, which are involved in psoriasis pathogenesis [50], were targeted by plasma miRNAs deregulated in psoriasis patients. The VEGF signaling pathway plays an important role in psoriasis [51]. Moreover, molecules associated with MAPK have been reported to be upregulated in the blood of psoriasis patients [52], and the p38 MAPK pathway is involved in psoriasis [53]. In addition, the WNT pathway is involved in skin inflammation in psoriasis patients [54].

The “proteoglycans in cancer” pathway was prominently targeted by deregulated plasma miRNAs of psoriasis patients. Although the pathway is typically associated with many types of cancers, proteoglycans are important in angiogenesis and in the migration and retention of leukocytes [39]. Target genes such as AKT2, WNT1, WNT4, WNT10, WNT11, FGF1, FGF7, FGF9, FGF11, and FGF14 are involved in inflammation, which is a typical feature of psoriasis. Thus, proteoglycan metabolism was significantly affected by deregulated plasma miRNAs in psoriasis.

To prioritize pathways that may be important for psoriasis pathogenesis, a pathway network analysis was performed. The network analysis indicated significant pathways in psoriasis pathogenesis, such as apoptosis, cell cycle, angiogenesis, inflammatory response, T cell immune response, VEGF, MAPK, WNT, JAK/STAT, NF-kappa B, and B cell response. Interestingly, pathways in the network were relevant to psoriasis pathogenesis. In addition, enriched pathways related to long-term depression, Alzheimer's disease, and glioma were detected.

Our data were associated with pathogenesis in psoriasis patients. We found that miR-214-3p, miR-7-5p, miR-761, miR-665, and miR-1207-5p showed the highest degree in the network analysis of deregulated plasma miRNAs and their target genes. Interestingly, some of the pathways were targeted by upregulated miRNAs, such as miR-7-5p and miR-761, whereas some of the pathways were targeted by downregulated miRNAs, such as miR-214-3p, miR-665, and miR-1207-5p.

It has been reported that psoriasis, a psychosomatic disease, is closely related to chronic stress (i.e., depression and anxiety) via the hypothalamic-pituitary-adrenal (HPA) axis, which secretes neuroendocrine mediators and triggers skin inflammation in psoriasis [55–57]. In addition, stress can induce the interaction between mast cells and microglia and lead to inflammation [58], and inflammation of the hypothalamus can also induce the secretion of neuroendocrine mediators and skin inflammation [59]. miR-214-3p plays an important role in depressive-like behaviours, cognition defects, Alzheimer's disease, and endothelial cell dysfunction and mediates neural apoptosis, neuropathic pain, and the growth, migration, and invasion of glioma cells [60–65]. miR-7-5p is relevant to vascular endothelial cell proliferation, glioma, brain damage, and cognitive dysfunction [66–69]. Moreover, it has been reported that miR-7-5p boosts apoptosis but inhibits cell proliferation and apoptosis of T lymphocytes, explaining the function of miR-214-3p and miR-7-5p in psoriasis [70, 71]. It was reported that miR-1207-5p may exert an inhibitory effect on VEGF [72]. We indicated that miR-214-3p and miR-7-5p can regulate the VEGF pathway by deregulating NFATC4, PIK3R3, PRKCB, and PIK3CD, and angiogenesis typically occurs in psoriatic lesions [1]. Upregulated miR-761 has been found to be involved in modulating the proliferation and migration of glioma cells and in inducing skin inflammation by the neuroendocrine system and HPA axis [73]. Downregulated miR-665 has been found to be involved in cognitive dysfunction and astrocyte and neuroblastoma cell growth [74–76].

The differentially expressed miRNAs we identified do not correspond to those of other studies in psoriasis, and a possible explanation of the discrepancy is that most of the previous studies used samples such as psoriatic lesion tissues, keratinocytes, hair roots/shaft, PBMCs, psoriasis epidermal cells, and dermal T cells, but not plasma [4, 21–24]. miRNA profiling in plasma samples may be quite different from miRNA profiling in local skin cells such as epidermal keratinocytes, Th17 cells, and PBMCs. miRNAs have been identified to be deregulated in (1) psoriatic lesion tissues (miR-203, miR-21, miR-146a, and miR-423), dermal inflammatory infiltrates of psoriatic skin (miR-142-3p, miR-193b, and miR-223), the stratified epidermis of psoriatic lesions (miR-135b and miR-150), the upper part of the epidermis (miR-99a and miR-150), and psoriatic skin of animal models of psoriasis (miR-424); (2) keratinocytes (miR-99a, miR-150, miR-423, and miR-197), keratinocytes in psoriatic lesions (miR-31, miR-203, and miR-125b), normal human keratinocytes stimulated by combinations of proinflammatory cytokines (miR-203), and the reconstituted human epidermis and human keratinocyte cell line stimulated by IL-22 (miR-184); (3) psoriasis epidermal cells and dermal T cells (miR-21), Th17 cells (miR-193b and miR-223), and CD4(+) T cells (miR-210); (4) psoriasis hair roots (miR-19a) and psoriasis hair shafts (miR-424); and (5) PBMCs (miR-193b, miR-223, miR-143, and miR-146a).

miR-1266 and miR-33 have been detected to be deregulated in blood samples of patients with psoriasis. miR-1266 is overexpressed in psoriasis serum samples [77], which may be differentially expressed in plasma. In addition, since miR-33 is involved in the posttranscriptional regulation of genes associated with lipid metabolism, one study reported that the plasma levels of lipid and glucose metabolism-related miR-33 are increased and correlated with insulin in psoriasis patients [78].

The potential limitations of the study are as follows: (1) We analysed deregulated miRNAs in the plasma samples from psoriasis patients, and the plasma miRNA profiles may be different than the miRNA profiles in skin tissue. (2) We identified the upregulation or downregulation of plasma miRNAs, and the changes in mRNA and gene expression were not confirmed. (3) Therapeutic interventions were not included in our study, so we cannot associate the expression of plasma miRNAs with the treatment responses; these experiments can be done in the future.

## 5. Conclusion

In summary, we studied the global plasma miRNA expression profile in psoriasis. Using this approach, we clarified specific plasma miRNA expression profiles that characterize psoriasis. The study analysed the expression of plasma miRNAs and the target pathways, elucidating the pathogenesis of psoriasis. This study illuminated some mechanisms of psoriasis pathogenesis by analysing deregulated plasma miRNAs, which may be used for designing novel therapeutic strategies and identifying diagnostic biomarkers for psoriasis.

## Figures and Tables

**Table 1:** Clinical characteristics of psoriasis patients.

Patients utilized for a gene array study	15 (100%)
Sex	
Male	9
Female	6
Age at diagnosis	36.80 ± 10.16
Types of patients	
Plaque psoriasis	15 (100%)
Psoriatic arthritis	0
Pustulosis of the palms and soles	0
Erythrodermic psoriasis	0
Stage	
Active stage	11 (73%)
Stable period	4 (27%)

**Table 2:** 

MicroRNA	Sequence	*P* value	Fold change (patients/control)	Regulation
(a) Plasma miRNAs upregulated in psoriasis patients versus healthy subjects				
hsa-miR-1271-5p	CUUGGCACCUAGCAAGCACUCA	0.0005695	3.31	Up
hsa-miR-299-5p	UGGUUUACCGUCCCACAUACAU	0.0006351	12.54	Up
hsa-miR-760	CGGCUCUGGGUCUGUGGGGA	0.001499	5.21	Up
hsa-miR-7-5p	UGGAAGACUAGUGAUUUUGUUGU	0.0022822	2.68	Up
hsa-miR-92b-3p	UAUUGCACUCGUCCCGGCCUCC	0.0045117	3.17	Up
hsa-miR-130b-5p	ACUCUUUCCCUGUUGCACUAC	0.0054175	2.43	Up
hsa-miR-510-5p	UACUCAGGAGAGUGGCAAUCAC	0.0155792	9.3	Up
hsa-miR-224-3p	AAAAUGGUGCCCUAGUGACUACA	0.015879	3.68	Up
hsa-miR-218-5p	UUGUGCUUGAUCUAACCAUGU	0.0167446	5.52	Up
hsa-miR-379-3p	UAUGUAACAUGGUCCACUAACU	0.0267967	3.13	Up
hsa-miR-761	GCAGCAGGGUGAAACUGACACA	0.033785	2.41	Up
hsa-miR-10b-3p	ACAGAUUCGAUUCUAGGGGAAU	0.0344881	4.04	Up
hsa-miR-624-5p	UAGUACCAGUACCUUGUGUUCA	0.0364547	2.74	Up
hsa-miR-577	UAGAUAAAAUAUUGGUACCUG	0.044155	3.05	Up
hsa-miR-935	CCAGUUACCGCUUCCGCUACCGC	0.0455152	4.76	Up
(b) Plasma miRNAs downregulated in psoriasis patients versus healthy subjects				
hsa-miR-665	ACCAGGAGGCUGAGGCCCCU	0.0003116	0.065	Down
hsa-miR-320d	AAAAGCUGGGUUGAGAGGA	0.0023854	0.42	Down
hsa-miR-10b-5p	UACCCUGUAGAACCGAAUUUGUG	0.0109296	0.43	Down
hsa-miR-1260a	AUCCCACCUCUGCCACCA	0.0121104	0.42	Down
hsa-miR-362-3p	AACACACCUAUUCAAGGAUUCA	0.0130663	0.49	Down
hsa-miR-214-3p	ACAGCAGGCACAGACAGGCAGU	0.0169923	0.37	Down
hsa-miR-30a-5p	UGUAAACAUCCUCGACUGGAAG	0.0170545	0.37	Down
hsa-let-7b-3p	CUAUACAACCUACUGCCUUCCC	0.0235985	0.45	Down
hsa-miR-638	AGGGAUCGCGGGCGGGUGGCGGCCU	0.0240685	0.36	Down
hsa-miR-320c	AAAAGCUGGGUUGAGAGGGU	0.0241893	0.39	Down
hsa-miR-504-5p	AGACCCUGGUCUGCACUCUAUC	0.0248942	0.21	Down
hsa-miR-100-5p	AACCCGUAGAUCCGAACUUGUG	0.0254685	0.36	Down
hsa-miR-765	UGGAGGAGAAGGAAGGUGAUG	0.0363717	0.24	Down
hsa-miR-887-3p	GUGAACGGGCGCCAUCCCGAGG	0.038367	0.23	Down
hsa-miR-1207-5p	UGGCAGGGAGGCUGGGAGGGG	0.0478966	0.13	Down

## Data Availability

The data used to support the findings of this study are available from the corresponding author upon request.
